# Supplementary figures and images for: ‘Necessity is the mother of invention’: Specialist palliative care service innovation and practice change in response to COVID-19. Results from a multinational survey (CovPall)
Source: Palliat Med. 2021 Mar 23;35(5):814–29. doi: 10.1177/02692163211000660 (PMC8114457; doi:10.1177/02692163211000660)

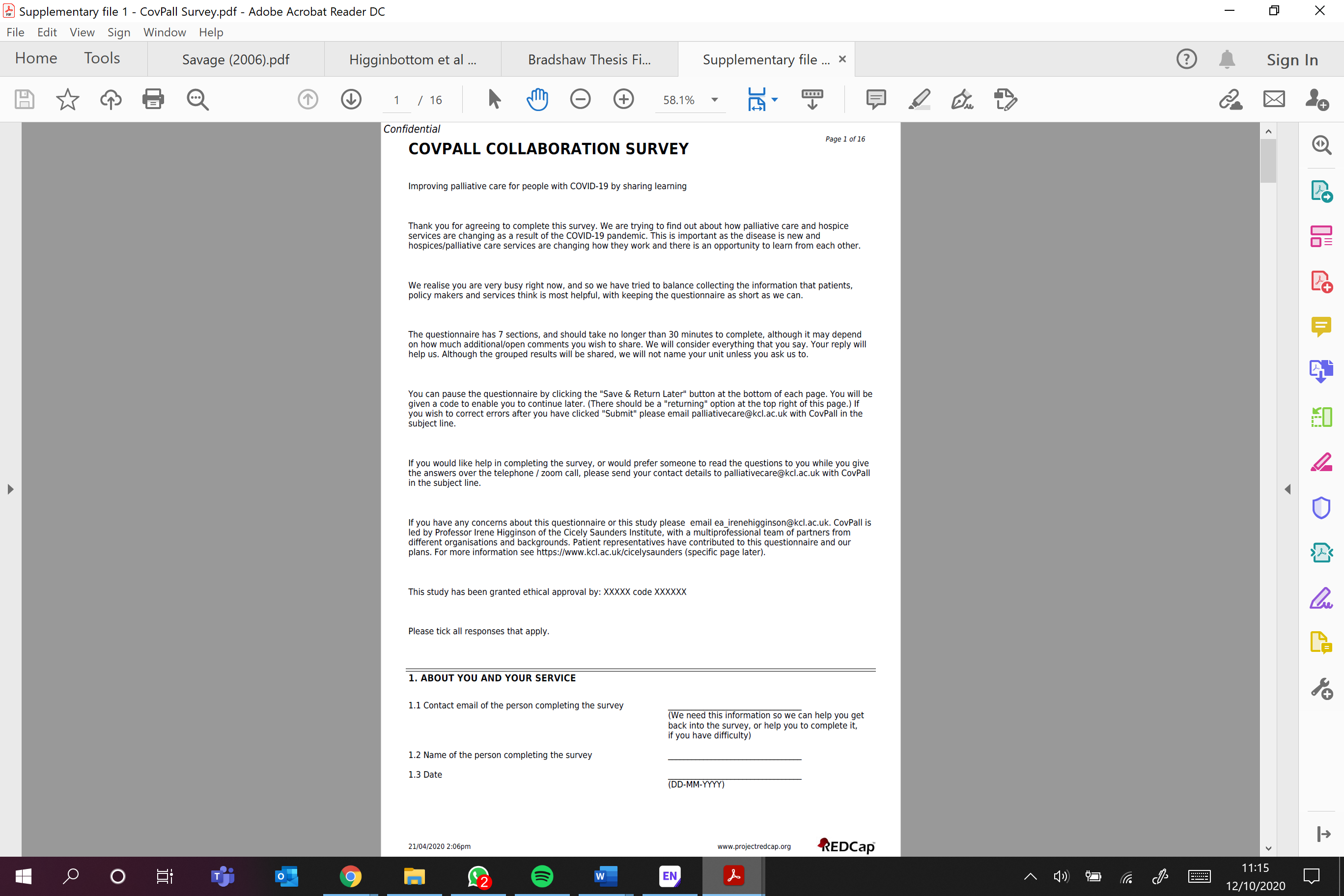


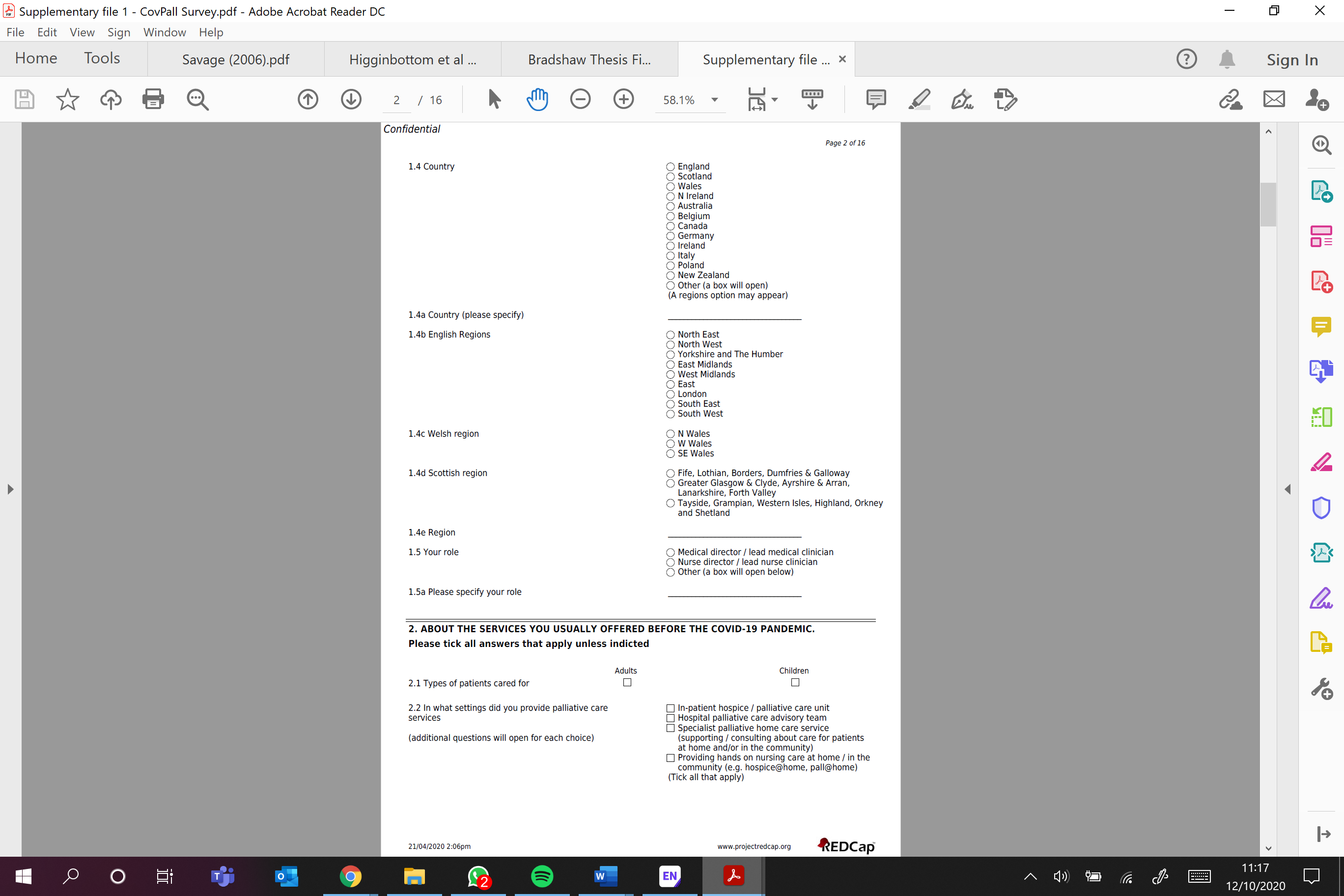


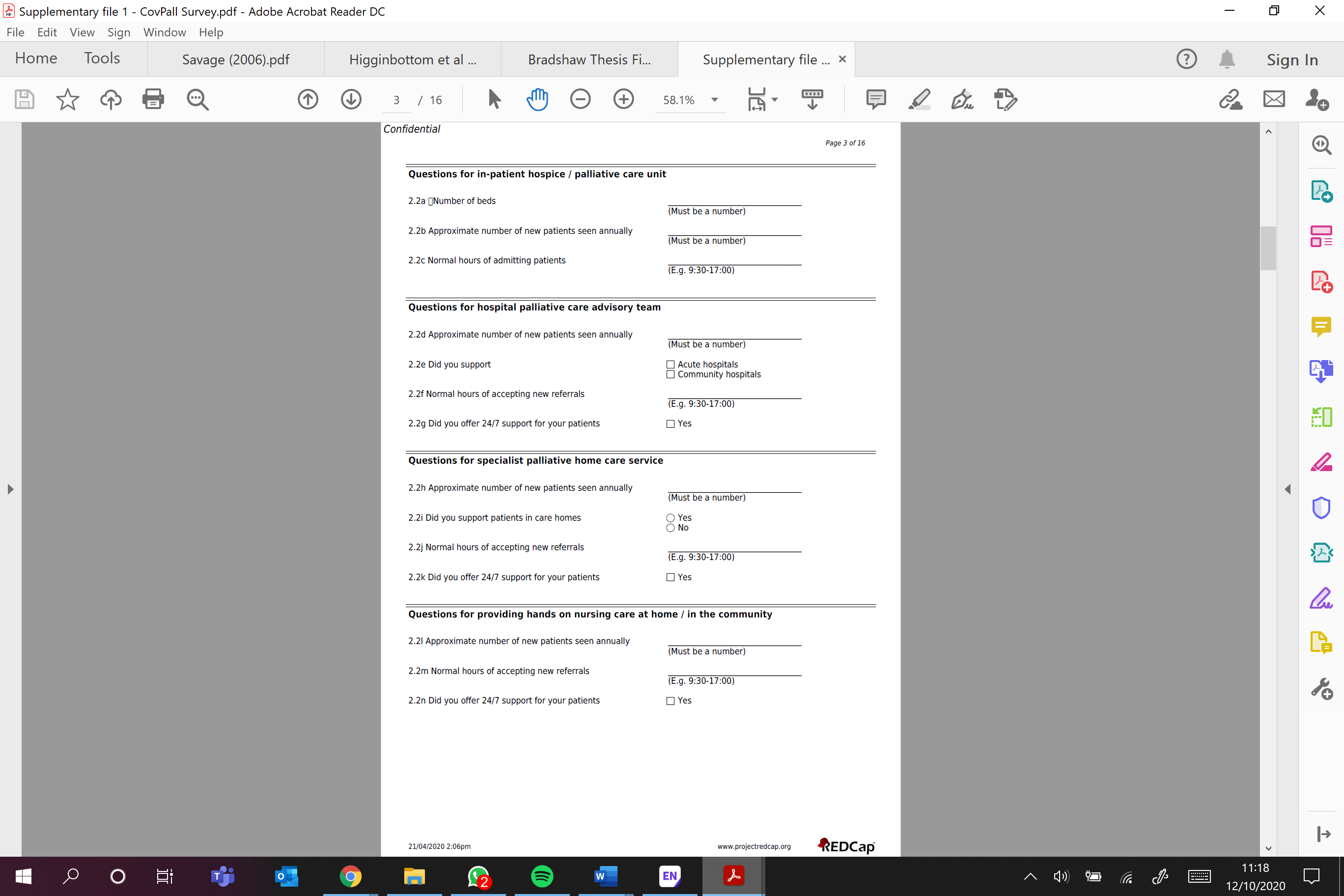


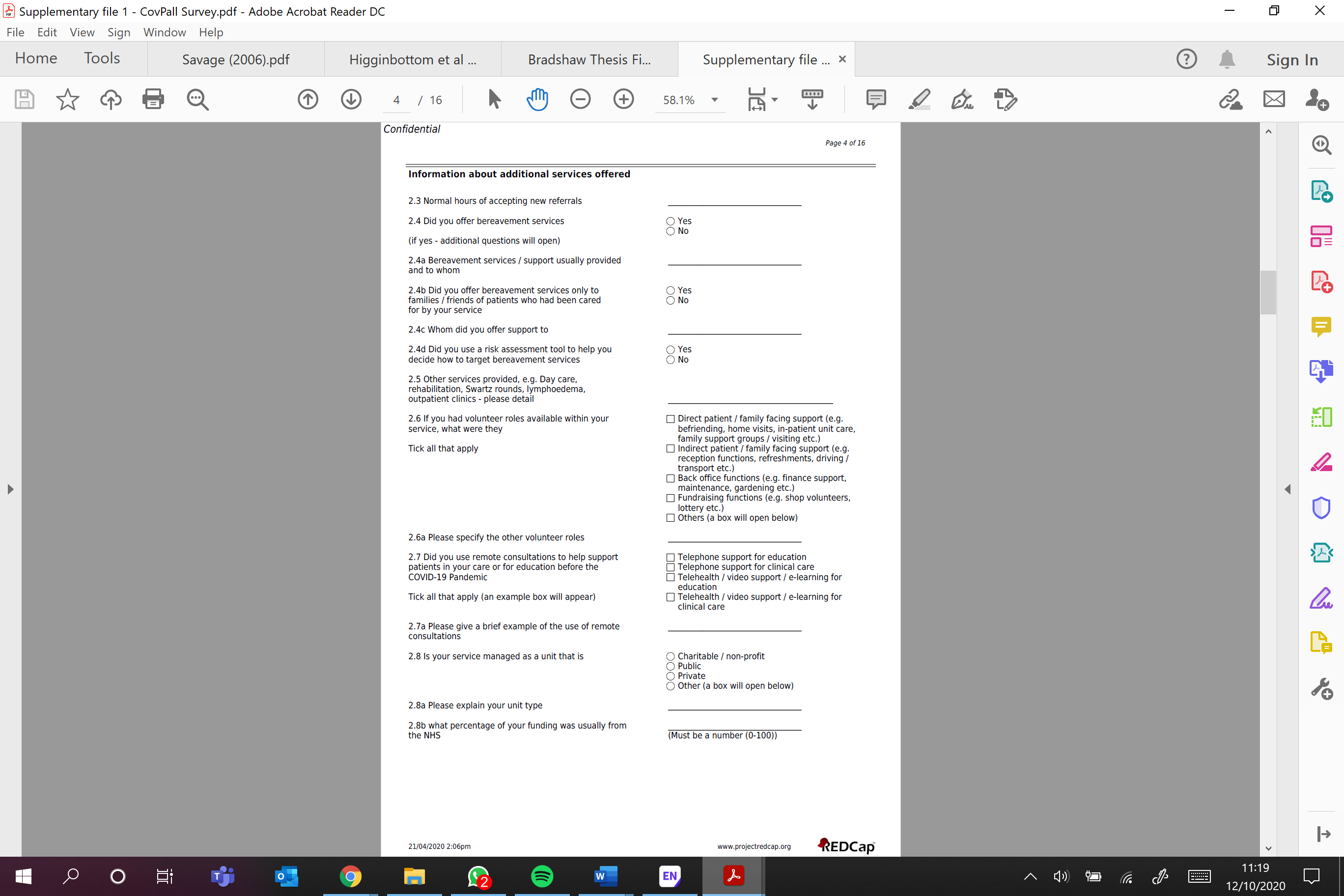


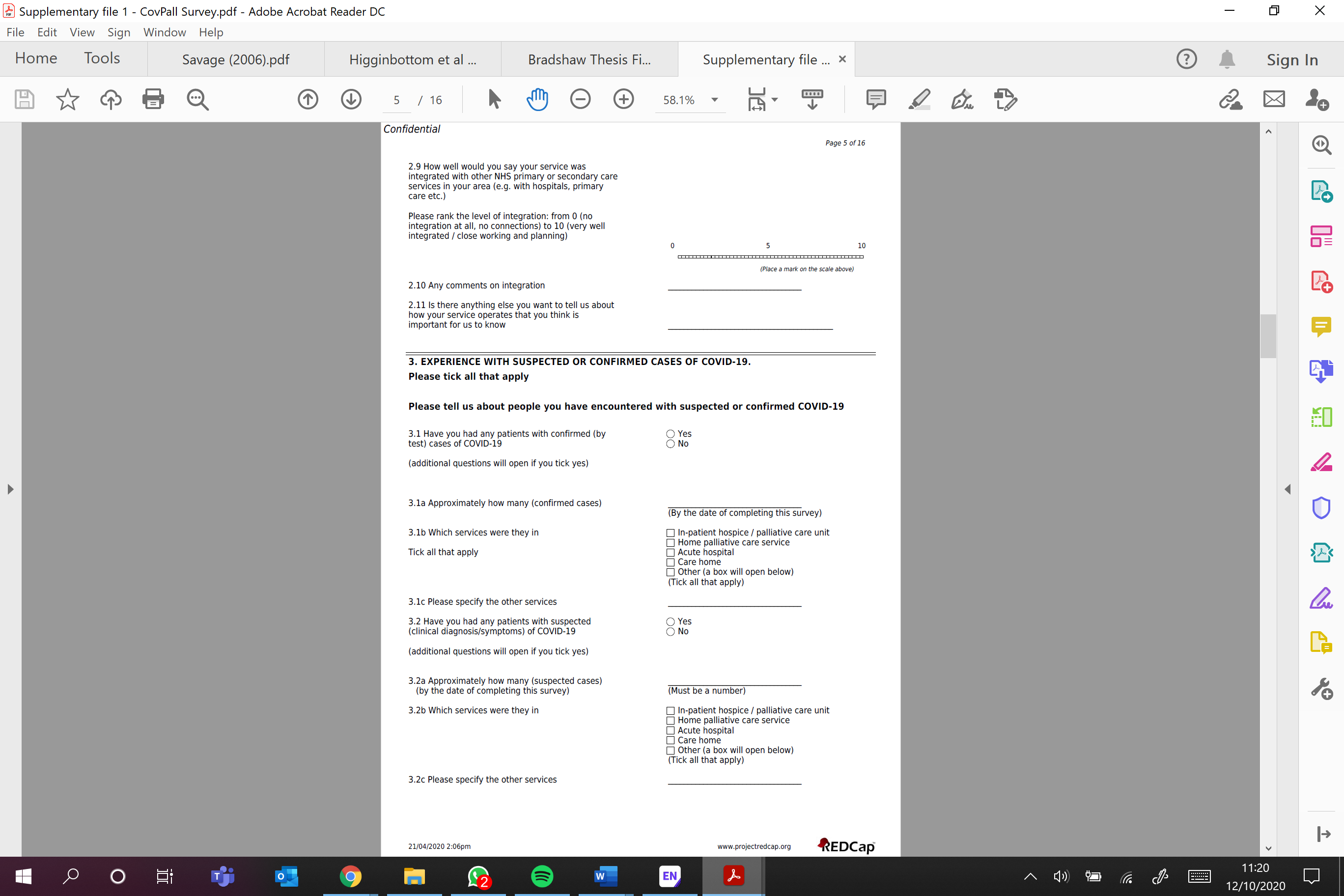


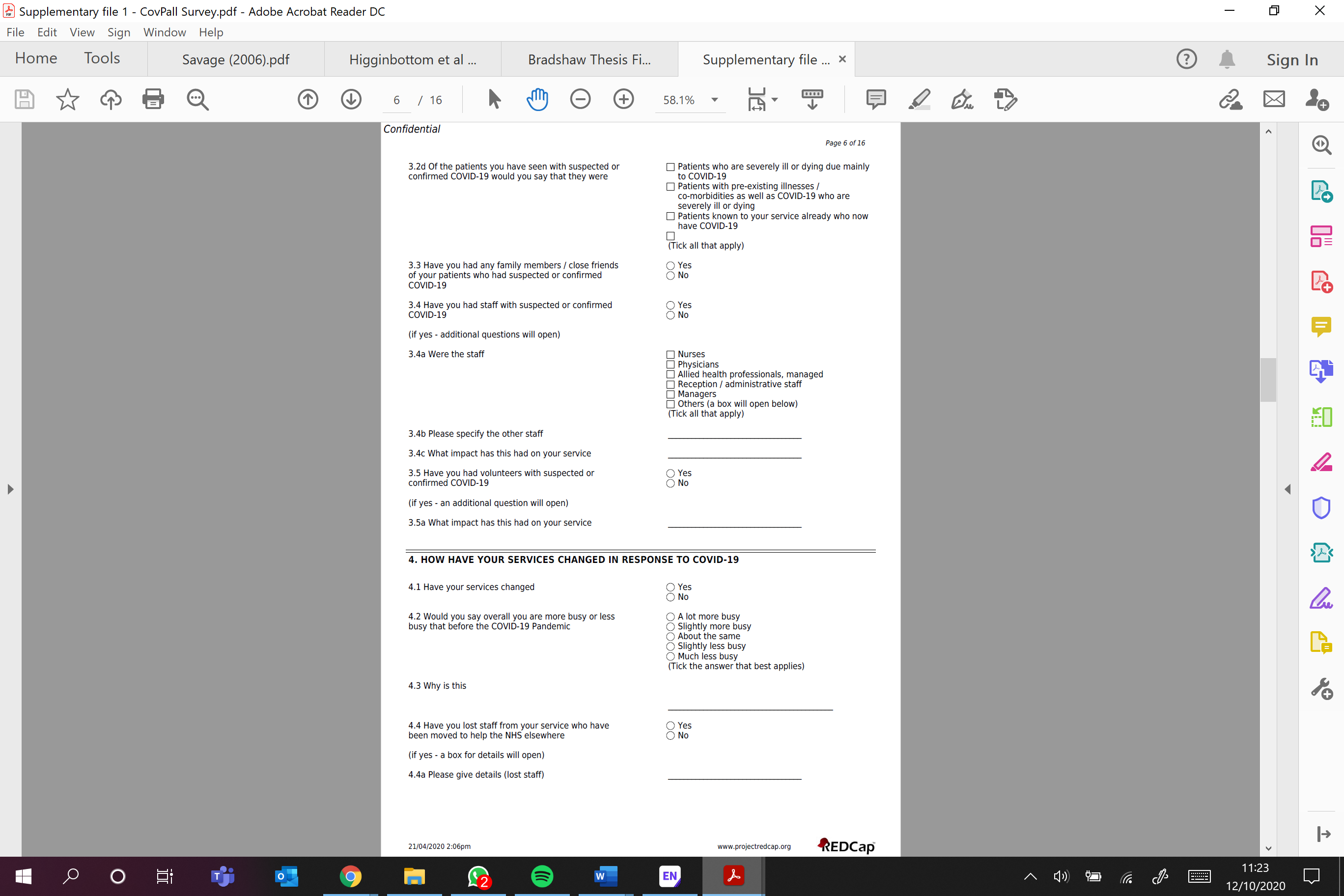


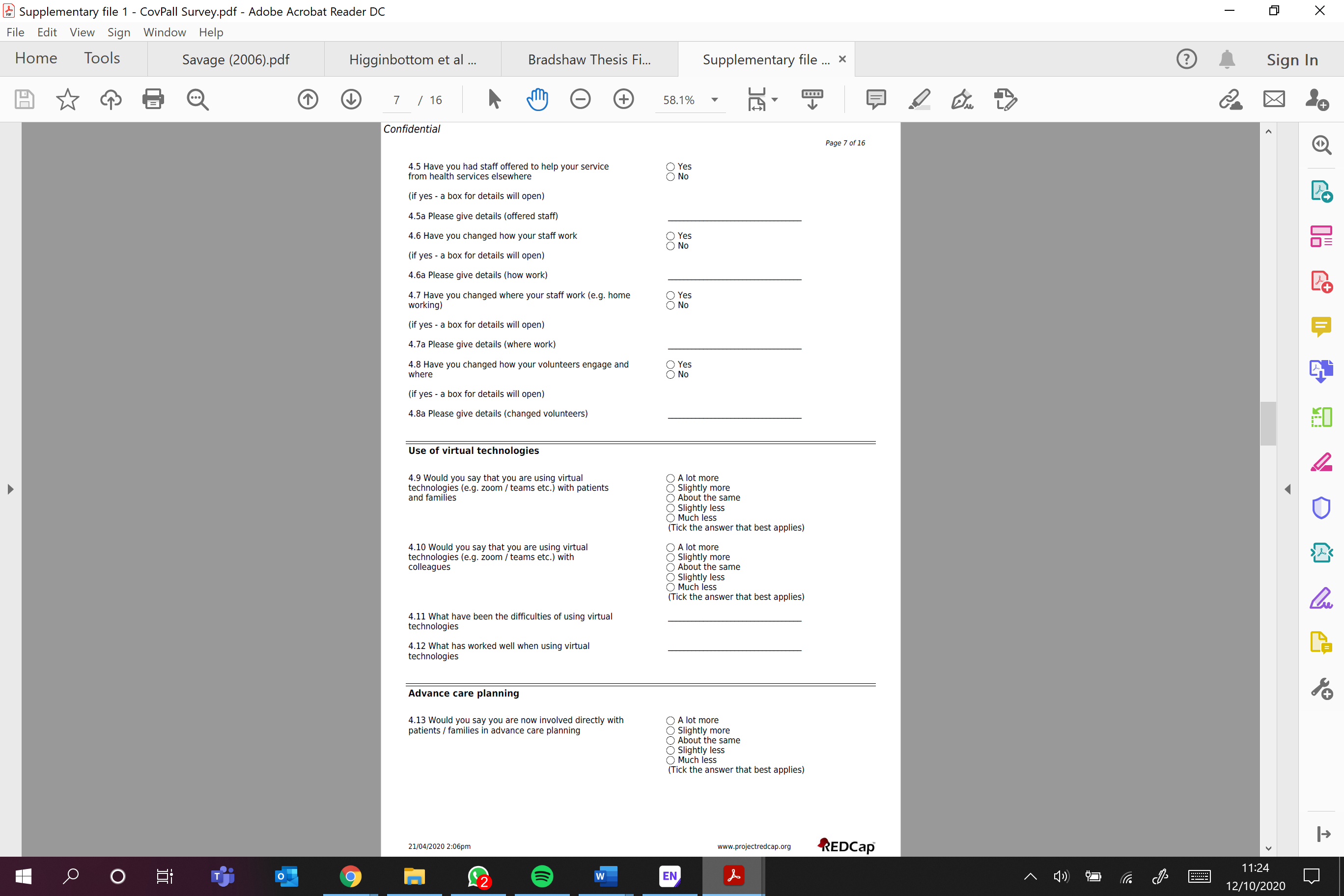


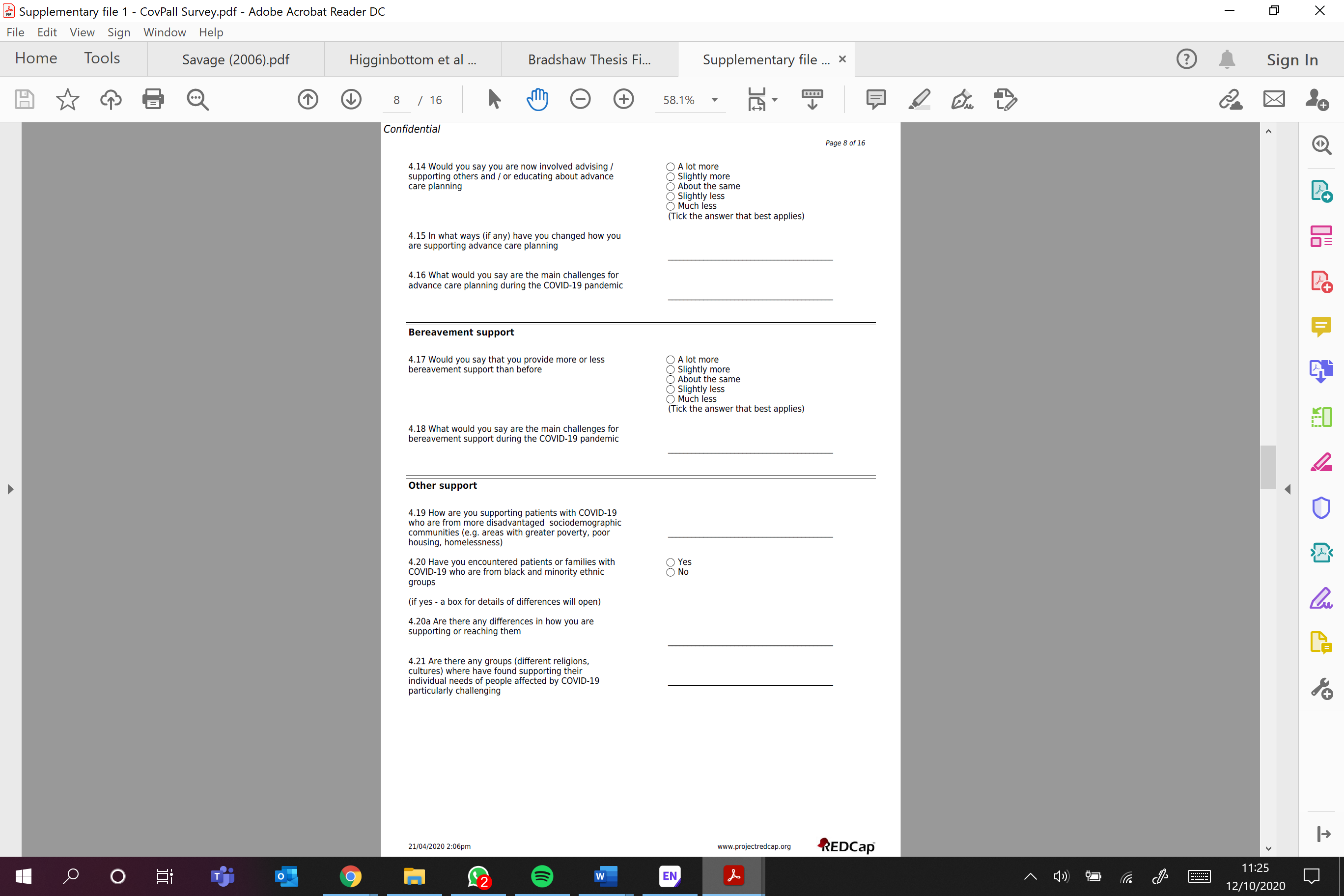


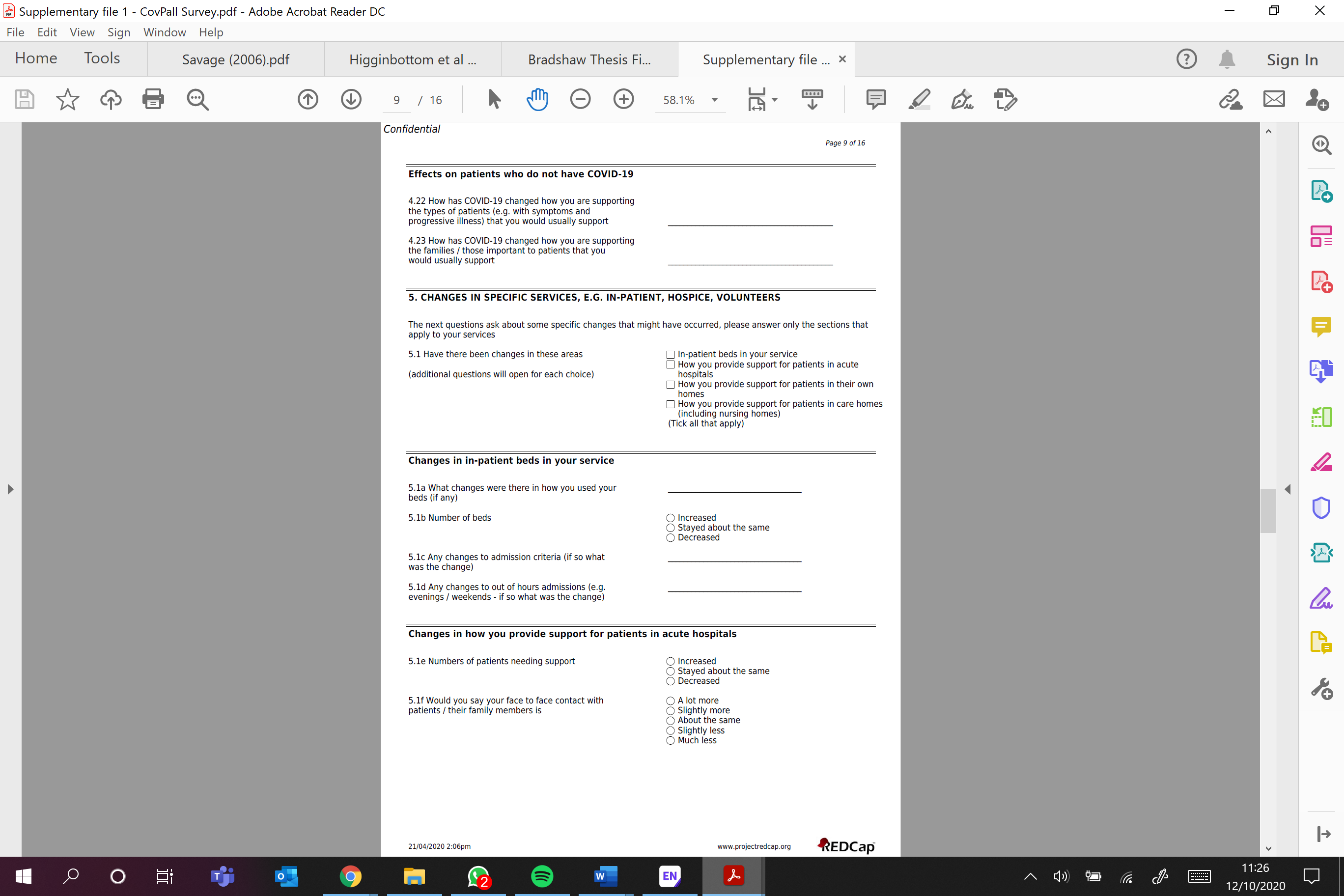


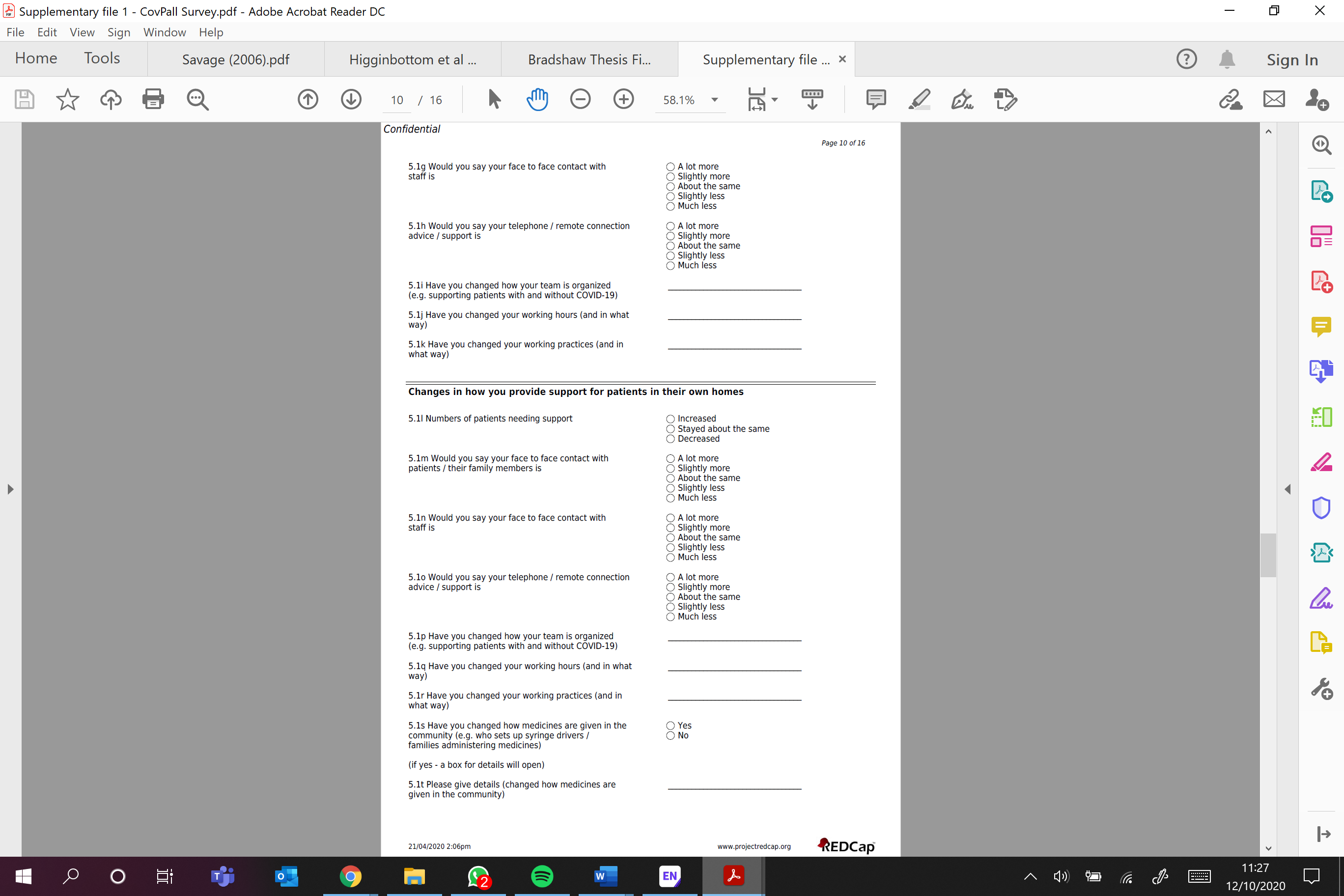


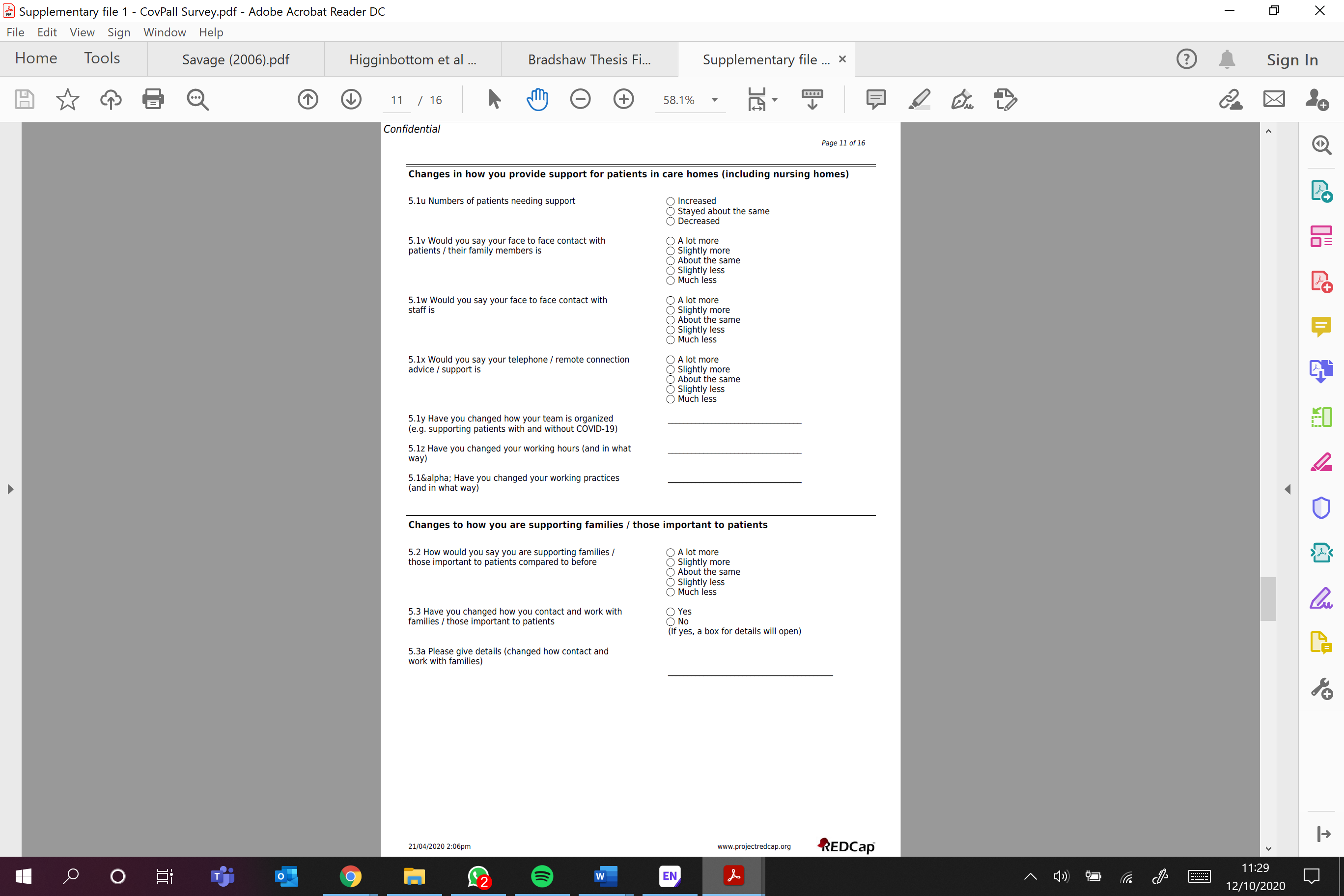


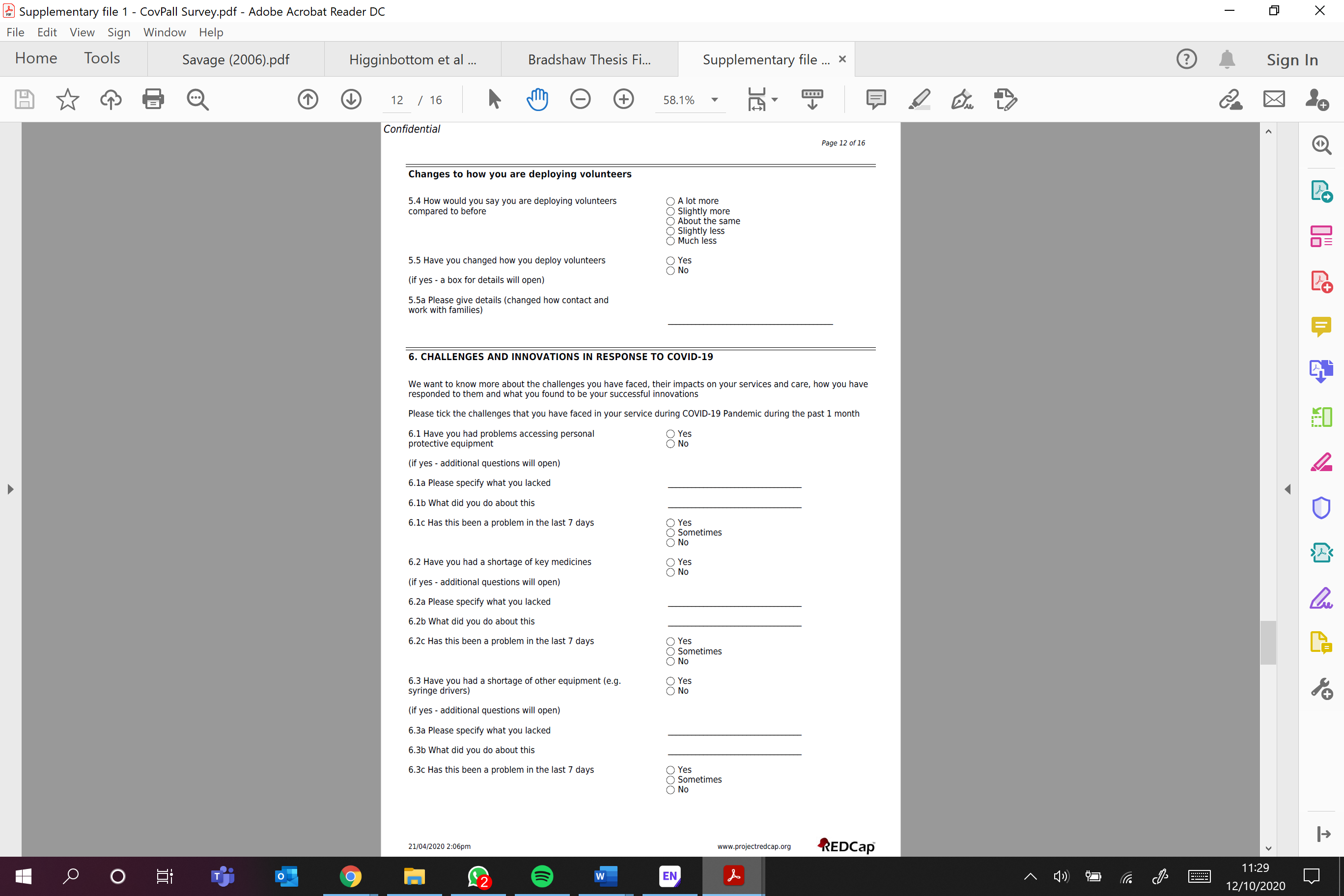


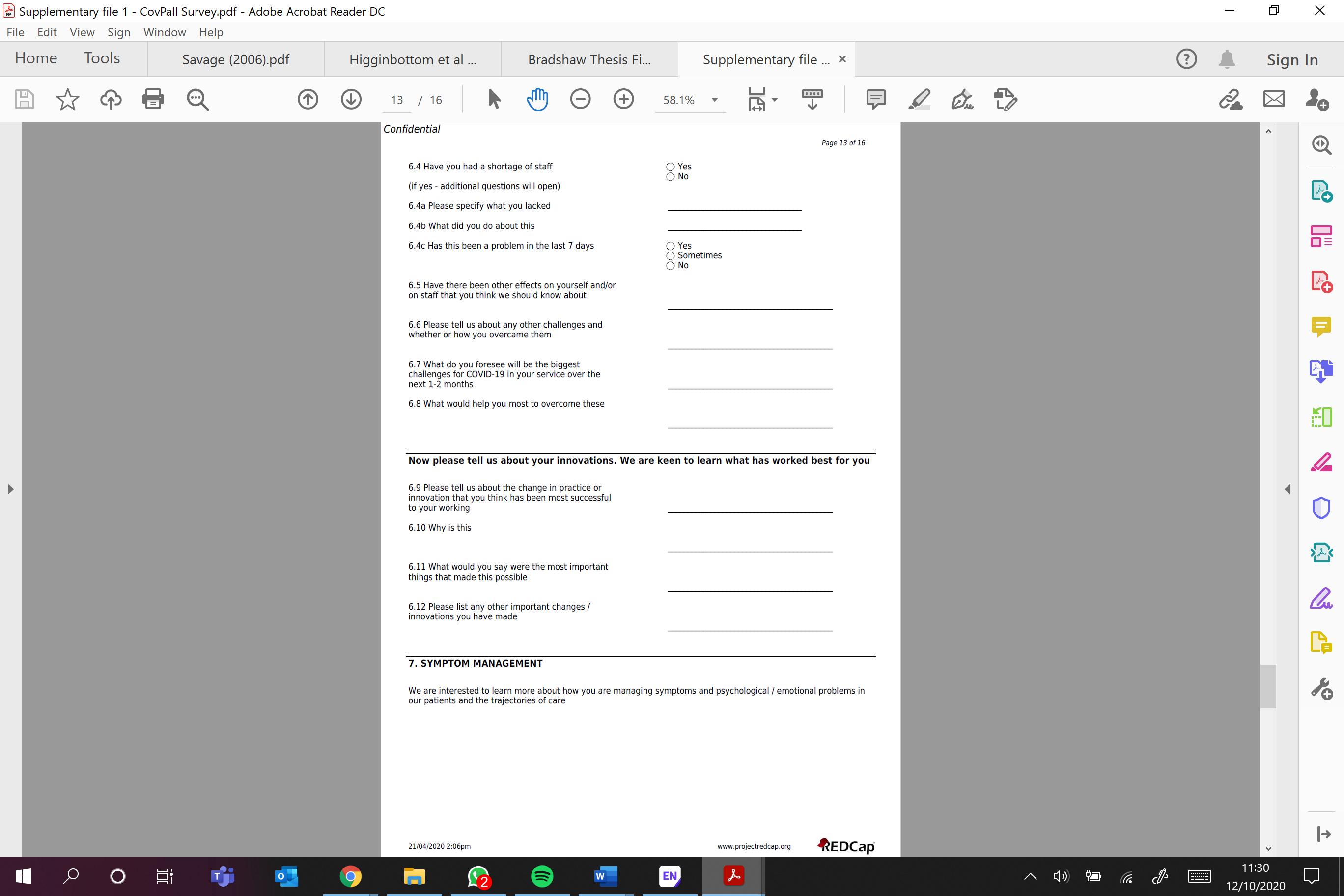


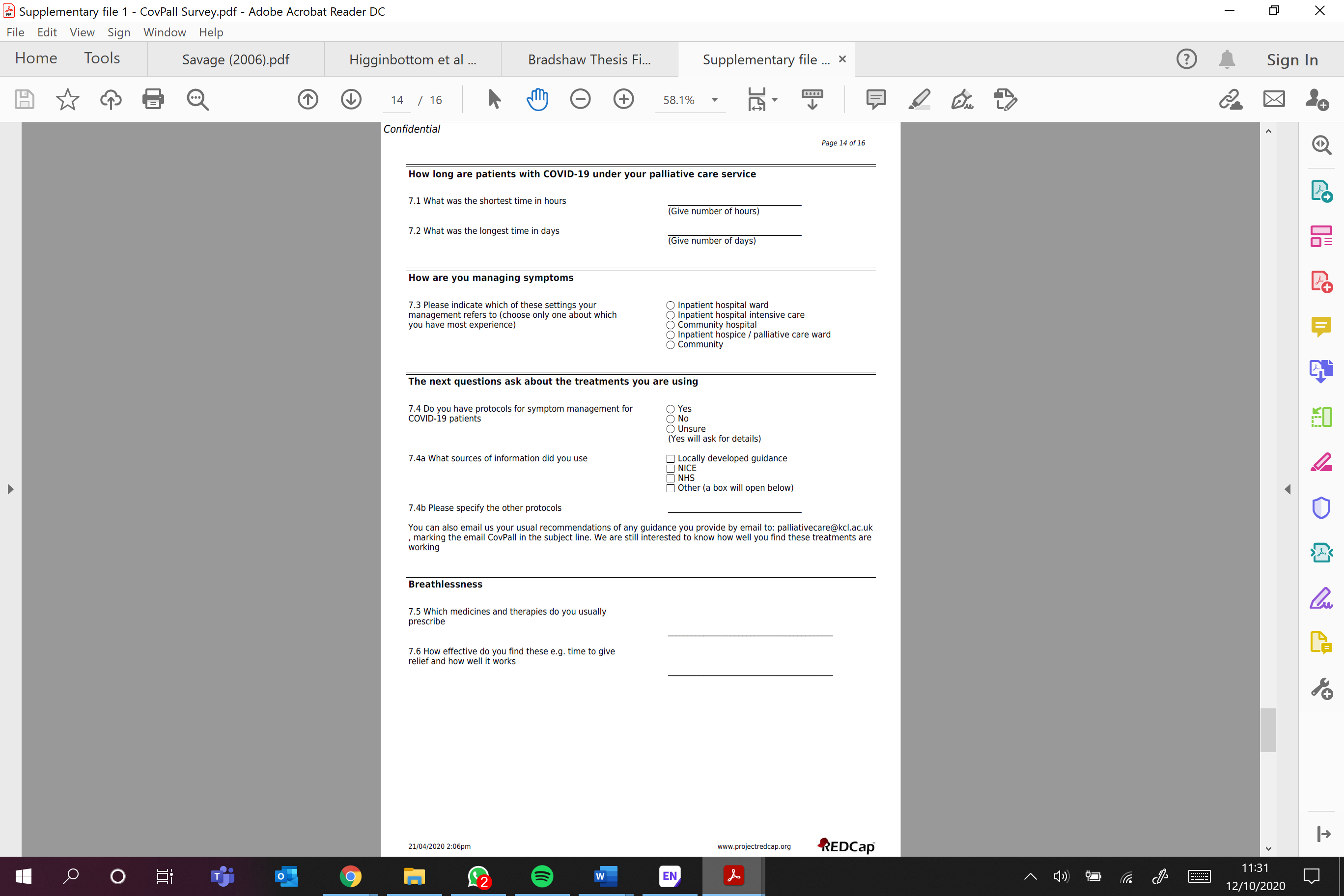


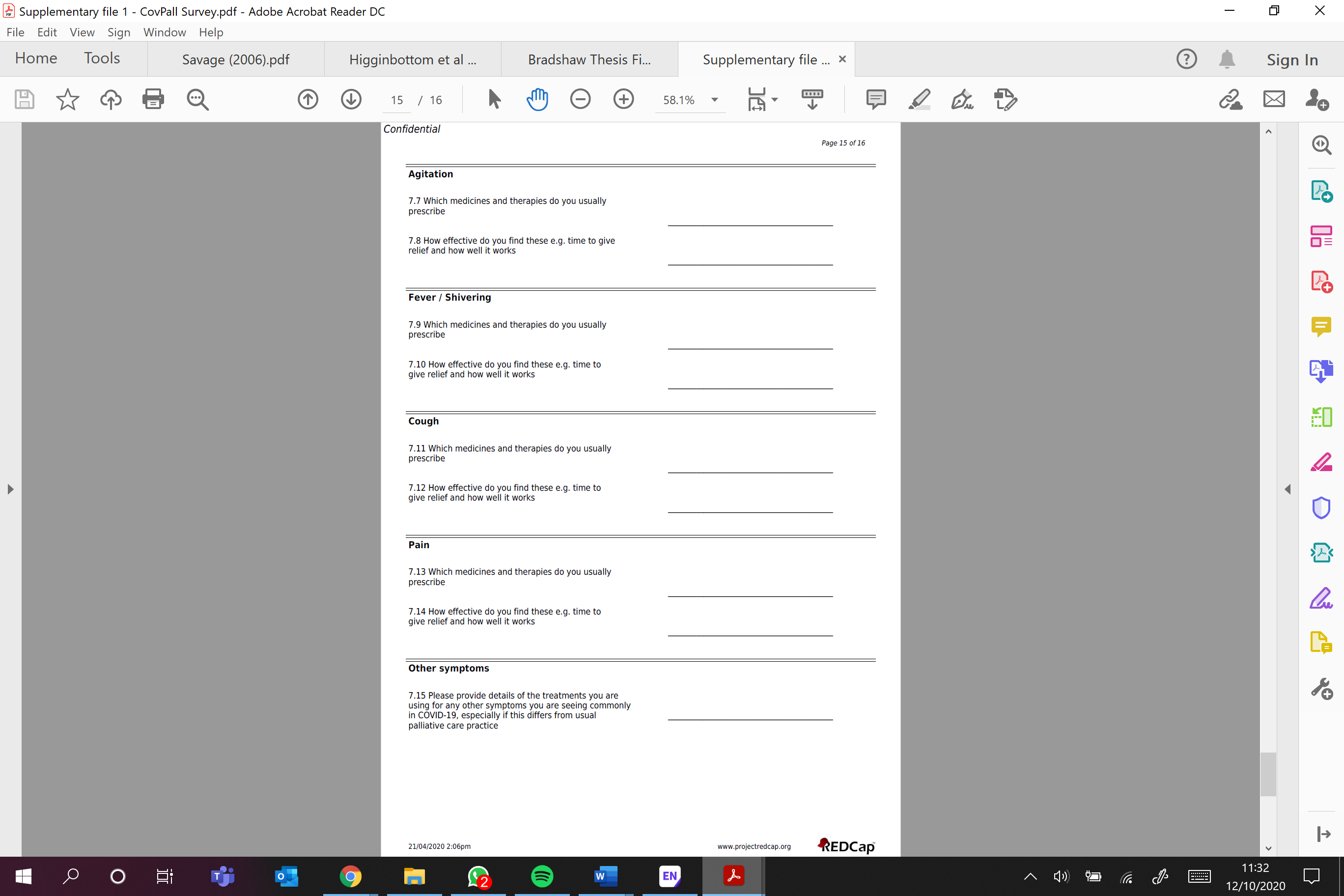


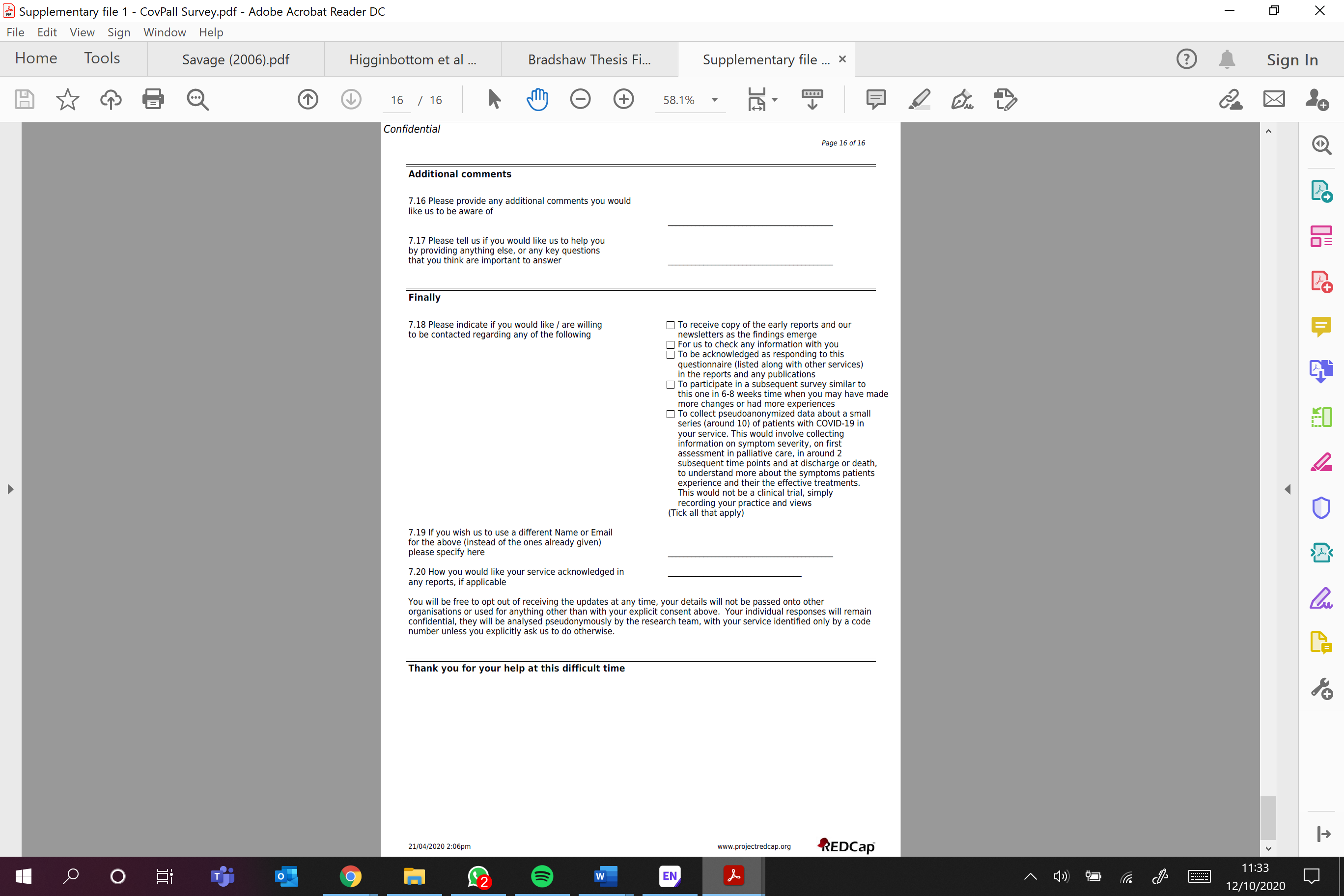

Supplement: sj-docx-1-pmj-10.1177_02692163211000660 – Supplemental material for ‘Necessity is the mother of invention’: Specialist palliative care service innovation and practice change in response to COVID-19. Results from a multinational survey (CovPall) [file sj-docx-1-pmj-10.1177_02692163211000660.docx]
