# Supplementary material for: ‘Necessity is the mother of invention’: Specialist palliative care service innovation and practice change in response to COVID-19. Results from a multinational survey (CovPall)
Source: Palliat Med. 2021 Mar 23;35(5):814–29. doi: 10.1177/02692163211000660 (PMC8114457; doi:10.1177/02692163211000660)
Supplement: sj-docx-3-pmj-10.1177_02692163211000660 – Supplemental material for ‘Necessity is the mother of invention’: Specialist palliative care service innovation and practice change in response to COVID-19. Results from a multinational survey (CovPall) [file sj-docx-3-pmj-10.1177_02692163211000660.docx]

**Supplementary file 3: Palliative care services provided in different settings**

| Palliative care and hospice services have multi-professional teams of dedicated staff trained in palliative care, comprising doctors, nurses, and often social workers and therapists. They provide expertise in pain and symptom management, holistic and psychosocial care, decision making, advance care planning, end of life care and often bereavement support. They include support in the following settings (one service may provide support in one or more setting):   1. Inpatient palliative care unit – provides specialist inpatient palliative care. It can be a ward within, or adjacent to, a hospital, or a free standing building. In some countries, it is called an inpatient hospice. 2. Hospital palliative care team – provides specialist palliative care advice and support to other clinical staff, patients and their families and carers in the hospital environment. They offer formal and informal education and liaise with other services in and out of the hospital. 3. Home palliative care team – provides specialist palliative care to patients who need it at home, or in care homes or residential homes, and support their families and carers. They also provide specialist advice to general practitioners, family doctors and nurses caring for the patients. 4. Home nursing – provides intensive home nursing care for the patient at home, sometimes referred to as hospice or hospital at home, often supporting patients whose care needs are such that without this they would be admitted to an inpatient palliative care unit or hospital. |
| --- |
