# Supplementary material for: ‘Necessity is the mother of invention’: Specialist palliative care service innovation and practice change in response to COVID-19. Results from a multinational survey (CovPall)
Source: Palliat Med. 2021 Mar 23;35(5):814–29. doi: 10.1177/02692163211000660 (PMC8114457; doi:10.1177/02692163211000660)
Supplement: sj-docx-4-pmj-10.1177_02692163211000660 – Supplemental material for ‘Necessity is the mother of invention’: Specialist palliative care service innovation and practice change in response to COVID-19. Results from a multinational survey (CovPall) [file sj-docx-4-pmj-10.1177_02692163211000660.docx]

**Supplementary file 4: Answers to the survey questions related to services offered pre Covid-19 explored in the CovPall innovation article.**

|  | **UK**  **(n = 277)** | **Europe (except UK)**  **(n = 85)** | **World (except Europe) (n = 95)** | | | **Total** |
| --- | --- | --- | --- | --- | --- | --- |
|  |  |  | **LIC/LMIC**  **(n = 17)** | **UMIC**  **(n = 19)** | **HIC**  **(n = 59)** |  |

| **Information about services offered before COVID-19 pandemic** | | | | | | |
| --- | --- | --- | --- | --- | --- | --- |
| **Use of remote consultations to help support patient care or education before COVID-19**  Telephone support for clinical care (n/N, %) | 179/277 (64.6%) | 54/85 (63.5%) | 13/17 (76.5%) | 11/19 (57.9%) | 45/59 (76.3%) | 303/458 (66.2%)* |
| **Use of remote consultations to help support patient care or education before COVID-19**  Telehealth/video support/e-learning for education (n/N, %) | 88/277 (31.8%) | 11/85 (12.9%) | 1/17 (5.9%) | 6/19 (31.6%) | 21/59 (35.6%) | 127/458 (27.7%)^+^ |
| **Use of remote consultations to help support patient care or education before COVID-19**  Telehealth/ video support/e-learning for clinical care (n/N, %) | 54/277 (19.5%) | 11/85 (12.9%) | 4/17 (23.5%) | 4/19 (21.1%) | 26/59 (44.1%) | 99/458 (21.6%)^+^ |

Note: UK = United Kingdom, LIC = Low Income Countries, LMIC = Lower Middle Income Countries, UMIC = Upper Middle Income Countries, HIC = High Income countries, PC = Palliative Care

# n of value and valid N denominator are provided. Percentages are of valid values, unless otherwise stated. Number of missing responses for each category are provided below. * Included data from the one missing country in the numerator and denominator. ^+^ Includes data from the one missing country in the denominator.
