# Supplementary material for: ‘Necessity is the mother of invention’: Specialist palliative care service innovation and practice change in response to COVID-19. Results from a multinational survey (CovPall)
Source: Palliat Med. 2021 Mar 23;35(5):814–29. doi: 10.1177/02692163211000660 (PMC8114457; doi:10.1177/02692163211000660)
Supplement: sj-docx-5-pmj-10.1177_02692163211000660 – Supplemental material for ‘Necessity is the mother of invention’: Specialist palliative care service innovation and practice change in response to COVID-19. Results from a multinational survey (CovPall) [file sj-docx-5-pmj-10.1177_02692163211000660.docx]

**Supplementary file 5: Table of Services to be acknowledged**

We would like to thank all the palliative care services and leads across the world for responding to the CovPall online survey.

The following persons and services indicated they were happy to be acknowledged for responding to the CovPall online survey.

| Alexander Devine Children’s Hospice Service |
| --- |
| Alison Wardrop District Nursing Sister, Hywel Dda University Health Board |
| Ashgate Hospicecare |
| Bangalore Baptist Hospital Palliative Care Service |
| BCUHB West HSPCT |
| Beatson West of Scotland Cancer Centre HSPCT |
| Beaumond House Community Hospice |
| Betsi Cadwaladr University Health Board SPCT |
| Birmingham St Mary’s Hospice |
| Bluebell Wood Children's Hospice |
| Bolton NHS Foundation Trust Specialist Palliative Care Team |
| Bury Community Specialist Palliative Care Team |
| Cambridge University Hospital NHS Foundation Trust Palliative Care Service |
| Carmarthenshire Specialist Palliative Care Service, Hywel Dda University Health Board |
| Center for Palliative Care, University Hospital Cologne, Germany |
| Cesta domů, home hospice |
| Chelsea and Westminster Hospital |
| CHFT Hospital SPCT |
| Children’s hospices across Scotland (CHAS) |
| City Hospice Cardiff |
| Claire House Children's Hospice |
| Clinique de Médecine Palliative, CHU de Lille, France |
| CNWL UCLH |
| Community based clinical nurse specialists |
| Community Specialist Palliative Care Team, St Ann's Hospice, Salford |
| Consultant Dr Paul Coulter, CNS Elizabeth Anderson and CNS Kellyann O'Neill, Palliative Care Team, Inverclyde Royal Hospital |
| Cornwall Hospice Care |
| Croydon Health Services Macmillan Specialist Palliative Care Team |
| Cwm Taf Morgannwg SPCT North |
| Department of Palliative Care, Homerton University Hospital NHS Foundation Trust |
| Department of Palliative care, Sheffield Teaching Hospitals NHS Foundation Trust |
| Department of Palliative Medicine, LMU Munich, Germany |
| Department of Palliative Medicine, Southern Sector, South East Sydney Local Health District, New South Wales, Australia |
| Deutsche PalliativStiftung |
| Dorothy House Hospice |
| Dove House Hospice, Hull |
| Dr Charles Daniels, Medical Director, St Luke’s Hospice Harrow and Consultant in Palliative Medicine, LNWHUT. |
| Dr Jonathan Downie, Consultant in Paediatric Palliative Medicine, Supportive and Palliative Care Team, Royal Hospital for Children, Glasgow |
| East Anglia's Children's Hospices (EACH) |
| East Cheshire Hospice |
| East Sussex Healthcare NHS Trust Supportive and Palliative Care Team |
| Eastern Health |
| Ed Dubland MD CCFP pc |
| Ellenor Adult Services |
| Ellenor Children’s Services |
| Ellenor Hospice, Coldharbour Road, Gravesend, Kent |
| Ellenor Inpatient Ward |
| Ellenor Wellbeing Service |
| Elvis J Miti for Community Palliative Care, UZIMA Project Ndanda, Mtwara, Tanzania. |
| ESNEFT specialist palliative care team |
| Family Support Team, Children's Hospices Across Scotland |
| Farleigh Hospice |
| Felicia Kontopidis, Journey Home Hospice |
| Fondazione Antea, Rome, Italy |
| Fondazione FARO, Turin, ITALY |
| Forest Holme Hospice, Poole Hospital NHS Foundation Trust |
| Forget Me Not Children's Hospice |
| Garden House Hospice Care |
| GHNHSFT Specialist Palliative Care Community team |
| Glasgow Royal Infirmary Hospital Specialist Palliative Care Team |
| Greenwich & Bexley Community Hospice |
| GSTT Community Palliative Care Team |
| Hambleton & Richmondshire SPCT |
| Hampshire Hospitals NHS Foundation Trust |
| Harrogate and District NHS Foundation Trust Palliative Care Team |
| Heart of Kent Hospice |
| Helen & Douglas House |
| Hospice De Wingerd (Charim Care Group) |
| Hospice Isle of Man |
| Hospice of St. Francis Berkhamsted, Herts |
| Hospice of The Good Shepherd, Chester |
| Hospice Waikato |
| Hospiscare Devon |
| Hospital Divina Providencia, El Salvador |
| Hospital Palliative Care Team, Kingston Hospital NHS Foundation Trust |
| Hospital Palliative Care Team, University Hospitals of North Midlands |
| Hospital Palliative Care team, University Hospital Southampton |
| Hospital Selayang Palliative Care Unit |
| Hospital Specialist Palliative care team |
| Hospital Specialist Palliative Care Team, University Hospitals of Leicester NHS Trust |
| Hull University Teaching Hospitals NHS Trust |
| Instituto de Investigaciones Médicas Alfredo Lanari, Universidad de Buenos Aires |
| Isabel Hospice |
| J Yeomans, St Richards Hospice (Worcester) |
| James Cook University Hospital Acute SPCT |
| Thitima Phosri |
| John Taylor Hospice |
| Helen Hubert, Palliative care and End of Life Care Pharmacist, St Richard’s Palliative Care Team |
| Katharine House Hospice Banbury |
| Kauniala Hospital |
| Kenelm F McCormick M.D., Akron, Ohio |
| Kilbryde Hospice, South Lanarkshire |
| Kim Jones, Deputy Head of Clinical Services at Hospice of the Valleys |
| King's College Hospital NHS Foundation Trust |
| Klinik Susenberg, Zürich |
| Knysna Sedgefield Hospice |
| Lanarkshire SPC service |
| Laois Offaly Palliative Care Service Cloneygowan Co Offaly |
| Leeds Teaching Hospitals NHS Trust Palliative Care Team |
| Limewood Dementia Service, Stafford |
| London Northwest Healthcare University Trust |
| LOROS Hospice, Leicester |
| Lynn Bushor, Department of Veteran's Affairs |
| Macmillan Unit / Royal Bournemouth & Christchurch |
| Manchester Foundation Trust - MRI |
| Manchester Foundation Trust WTWA |
| Maricruz Macias Montero, Hospital General de Segovia, Seccion de Geriatria. |
| Marie Curie Community Nursing Service London |
| Marie Curie Hospice, Cardiff and the Vale |
| Marie Curie Hospice West Midlands |
| Marie Curie Hospice, Hampstead |
| Marie Curie Services in Lothian |
| Martlets Hospice |
| Martlets Hospice, Brighton |
| Maura Farrell Miller, Director, Hospice and Palliative Care Program, Department of Veterans Affairs Medical Center, Florida |
| Mellannorrlands Hospice Sundsvall Sweden |
| Michael Sobell Hospice and Harlington Hospice |
| Mid Cheshire Hospital NHS Trust Specialist Palliative Care Team |
| Midhurst Macmillan Service (Sussex Community NHS Foundation Trust) |
| Municipal Hospital Dr Cornel Igna, Palliative Care Department from Campia Turzii |
| Myton Hospice |
| Newham University Hospital Specialist Palliative Care Team - Barts Health NHS Trust |
| NHS Ayrshire & Arran Supportive Care Team |
| NHS Fife Specialist Palliative Care Service |
| NHS Grampian Specialist Palliative Care Team |
| NHS Specialist Palliative Care Unit |
| Nightingale House Hospice, North Wales |
| Ninewells Hospital Palliative Care Team |
| North London Hospice |
| Northern Ireland Hospice Adult Service |
| Northumbria Healthcare NHS Foundation Trust |
| Nottingham University Hospitals NHS Trust |
| Ofra Fried, Palliative Care Specialist, Townsville University Hospital, Queensland |
| Overgate Hospice |
| Palliativa Care Unit. west health Area. Valladolid. Castilla y León .Spain |
| Palliative and End of Life care Team, Newcastle upon Tyne Hospitals NHS Foundation Trust |
| Palliative Care Department, Bangalore Baptist Hospital |
| Palliative Care Service, Chaim Sheba Medical Center, Ramat-Gan, Israel |
| Palliative Care Service, Gloucestershire Hospitals NHS Foundation Trust |
| Palliative Care Team-Bassett Medical Center, Cooperstown NY |
| Palliative Care Team, Centre for Pain Management and Palliative Care, Haukeland University Hospital, Bergen, Norway |
| Palliative Care Unit, Bolzano, Italy |
| Palliative Care Unit, Bassini Hospital, Cinisello Balsamo, Milan, Italy |
| Palliative Care, Kantonsspital Olten, Switzerland |
| Palliative Medicine, UHDB NHS Trust |
| Palliaviva |
| Peace Hospice Care |
| Pembrokeshire Specialist Palliative Care service, Hywel Dda University Health Board |
| Phyllis Tuckwell Hospice Care |
| Pilgrims Hospices in East Kent |
| Pippa Hawley, Medical Director, Pain and Symptom Management Palliative Care, BC Cancer. |
| Portsmouth Hospital NHS Trust Palliative Care Service |
| Princess Alice Hospice |
| Prof Olaitan A Soyannwo |
| Queen Elizabeth University Hospital Glasgow Specialist Palliative Care Team, and Prince and Princess of Wales Hospice Glasgow |
| Queenscourt Supportive & Specialist Palliative Care Service |
| Rotherham NHS Foundation Trust Palliative Care Team |
| Rowans Hospice |
| Rowcroft Hospice |
| Royal Berkshire Foundation Trust Hospital Palliative Care Team |
| Royal Trinity Hospice |
| Sabar Health Home Hospital |
| Saint Francis Hospice |
| Salford Royal Hospital Specialist Palliative Care Team |
| Salisbury Specialist Palliative Care service |
| SAPV des OSP Karlsruhe |
| SCCU SEVILLA SUR, SAS. SPAIN. |
| Sengkang General Hospital, Singapore |
| Shooting Star Children's Hospice |
| Sobell House, Oxford University Hospitals |
| South East Palliative Care Services |
| South East Palliative Care, University Hospital Waterford, Ireland |
| South Tees Community Specialist Palliative Care MRC |
| South Tipperary Hospice Homecare Team |
| South Tyneside District Hospital Specialist Palliative Care Team, South Tyneside and Sunderland NHS Foundation Trust |
| Specialist Palliative Care Hospital Team. East Kent University Foundation Trust |
| Specialist Palliative Care Service South Eastern Health and Social Care Trust |
| Specialist Palliative Care Team Imperial College Healthcare NHS Trust |
| Specialist Palliative Care Team, Coventry and Warwickshire Partnership NHS Trust |
| Specialist Palliative Care Team, West Middlesex University Hospital, Chelsea and Westminster NHSFT |
| Specialist Palliative Care Service, County Durham and Darlington NHS Foundation Trust |
| St Ann's Hospice |
| St Barnabas Hospice, Lincolnshire |
| St Barnabas House, Worthing |
| St Bartholomew’s Hospital, Barts Health |
| St Catherine's Hospice, Preston |
| St Christopher's |
| St Clare Hospice, Essex |
| St Columba's Hospice, Edinburgh |
| St Gemma's Hospice - Leeds, West Yorkshire. |
| St Helena |
| St John of God Murdoch Hospital |
| St John's Hospice, Doncaster, RDASH |
| St Johns Hospice, Lancaster |
| St Leonard's Hospice, York |
| St Luke's Cheshire Hospice |
| St Luke's Combined Hospices, Cape Town, South Africa |
| St Luke's Hospice, Sheffield. |
| St Luke’s Hospice, Kenton, Harrow covering London boroughs of Brent and Harrow. Ursula Reeve, Director of Patient Services. Cathy Hanrott, Hospice Service Navigator |
| St Margaret’s Hospice, Somerset. |
| St Martha's Hospital Palliative Care Team |
| St Mary's Hospice, Ulverston, Cumbria |
| St Michael's Hospice, Hastings and Rother |
| St Oswald's Hospice, Newcastle |
| St Peter's Hospice |
| St Richards Hospice |
| St Wilfrid's Hospice, Chichester |
| St Wilfrid's Hospice, Eastbourne. |
| St. Luke's Hospice Community Services South West Essex |
| Stockport Specialist Palliative Care Service |
| Strathcarron Hospice |
| Sue Ryder Leckhampton Court Hospice |
| Sue Ryder Manorlands Hospice |
| Susanna Sandöy, Capio Asih Nacka, |
| Swansea Bay University Health Board Specialist Palliative Care Service (which includes Ty Olwen Hospice) |
| Symptom Control and Palliative Care Team, Royal Marsden NHS Foundation Trust |
| Tatiana Chavouzi |
| Tayside Palliative Care Service |
| The Arthur Rank Hospice, Cambridge |
| The Camden, Islington ELiPSe Palliative Care Team |
| The Hillingdon Hospital Palliative Care team |
| The Prince of Wales Hospice, Pontefract |
| The Wisdom Hospice and Medway Community Healthcare |
| TOPAT Zuyderland MC |
| Trinity Hospice & Brian House Children's Hospice |
| Trinity Hospice, Blackpool and Blackpool Teaching Hospitals |
| Tynedale Hospice at Home |
| Unità di Cure Palliative - Hospice Casale Monferrato ASL AL Piemonte |
| University Hospital Lewisham Macmillan Specialist Palliative Care Team, Lewisham and Greenwich NHS Trust |
| University Teaching Hospital Lusaka Adult Medicine Palliative Care Unit |
| Waterford Hospice Homecare Team |
| Weldmar Hospicecare |
| West Suffolk Hospital NHS Foundation Trust |
| Western Australia Paediatric & Adolescent Palliative Oncology, Perth Children's Hospital |
| Weston Hospicecare |
| Whittington Health Palliative Care Service |
| Wigan & Leigh Hospice |
| Wrighinton, Wigan and Leigh NHS Teaching Trust (Supportive and Palliative Care Team) |
| York Teaching Hospital Foundation Trust |
| York Teaching Hospitals |
| Zuercher Lighthouse |
| Zuzana Kremenova |
